# No evidence that spice consumption is a cancer prevention mechanism in human populations

**DOI:** 10.1093/emph/eoac040

**Published:** 2022-11-24

**Authors:** Antoine M Dujon, Aurélie Tasiemski, Pascal Pujol, Anthony Turpin, Beata Ujvari, Frédéric Thomas

**Affiliations:** Centre for Integrative Ecology, Geelong, School of Life and Environmental Sciences, Deakin University, Waurn Ponds, VIC 3216, Australia; CANECEV-Centre de Recherches Ecologiques et Evolutives sur le Cancer (CREEC), Montpellier 34090, France; CREEC/(CREES), MIVEGEC, Unité Mixte de Recherches, IRD 224-CNRS 5290-Université de Montpellier, Montpellier, France; Université de Lille, CNRS, Inserm, CHU Lille, Institut Pasteur de Lille, U1019 - UMR 9017 - CIIL - Center for Infection and Immunity of Lille, F-59000 Lille, France; CANECEV-Centre de Recherches Ecologiques et Evolutives sur le Cancer (CREEC), Montpellier 34090, France; Centre Hospitalier Universitaire Arnaud de Villeneuve, Montpellier, France; Institut Pasteur de Lille, UMR9020-UMR-S 1277-Canther-Cancer Heterogeneity, Plasticity and Resistance to Therapies, CNRS, Inserm, CHU Lille, Université de Lille, Lille, France; Medical Oncology Department, CHU Lille, University of Lille, Lille, France; Centre for Integrative Ecology, Geelong, School of Life and Environmental Sciences, Deakin University, Waurn Ponds, VIC 3216, Australia; CANECEV-Centre de Recherches Ecologiques et Evolutives sur le Cancer (CREEC), Montpellier 34090, France; CANECEV-Centre de Recherches Ecologiques et Evolutives sur le Cancer (CREEC), Montpellier 34090, France; CREEC/(CREES), MIVEGEC, Unité Mixte de Recherches, IRD 224-CNRS 5290-Université de Montpellier, Montpellier, France

**Keywords:** behaviour, prophylaxy, oncogenes

## Abstract

**Background:**

Why humans historically began to incorporate spices into their diets is still a matter of unresolved debate. For example, a recent study (Bromham et al. There is little evidence that spicy food in hot countries is an adaptation to reducing infection risk. *Nat Hum Behav* 2021;5:878–91.) did not support the most popular hypothesis that spice consumption was a practice favoured by selection in certain environments to reduce food poisoning, parasitic infections, and foodborne diseases.

**Methods:**

Because several spices are known to have anticancer effects, we explored the hypothesis that natural selection and/or cultural evolution may have favoured spice consumption as an adaptive prophylactic response to reduce the burden of cancer pathology. We used linear models to investigate the potential relationship between age-standardized gastrointestinal cancer rates and spice consumption in 36 countries.

**Results:**

Patterns of spice are not consistent with a cancer mitigation mechanism: the age-standardized rate of almost all gastrointestinal cancers was not related to spice consumption.

**Conclusions:**

Direction other than foodborne pathogens and cancers should be explored to understand the health reasons, if any, why our ancestors developed a taste for spices.

## INTRODUCTION

A spice is an ingredient added to a dish in relatively small quantities, primarily for flavour, colour or smell, rather than for bulk or nutrition (according of the definition of [1] and [2]). Spices are widely used across the globe and have been part of the diet of human populations for thousands of years. For instance, plant fossil evidence suggest garlic mustard seed (*Alliaria petiolata*) was used 6000 years ago as a spice in Europe [3] and that chilli peppers (*Capsicum* spp.) were widespread 6000 years ago in South America [4]. In 408 BCE Alaric the Goth besieged Rome and demanded 3000 pounds of pepper and various precious metal as ransom [5]. Ancient Egyptian papyri from 1555 BCE record the use of coriander, fennel, juniper, cumin, garlic and thyme [6]. The spatiotemporal extent of spice use therefore significantly overlaps with the evolutionary history of human societies over the past millennia [7].

Although spicy dishes are both widespread and common nowadays around the world, it remains unclear why humans historically started to incorporate spices in their food. A common hypothesis is that consuming spices provided an evolutionary advantage by being protective against pathogens such as digestive parasites or bacteria [7, 8]. For instance, Sherman and Billing suggested that spices reduce the risk of contracting food borne diseases because they contain phytomolecules with antibacterial and/or anti-parasitic properties. Thus, adding spice to a dish would reduce the risk of food poisoning, parasitic infection and foodborne illness, especially in hot countries where food spoils fast, refrigeration was only recently introduced and where oncogenic parasites are abundantly present [1, 5, 9]. This hypothesis was supported by a strong correlation between the mean number of spicy ingredients used in a recipe and the average temperature of a given country, especially for meats and, to a lesser extent, for vegetables, which are susceptible to spoilage [1, 5, 9]. However, Bromham *et al.* [2] recently demonstrated that this correlation was mostly explained by spatial auto-correlation effects, i.e. closely related countries tend to have similar culture and cuisines. When taking in account the lack of independence between countries in their analyses, they found that spices’ use was better reflected by global patterns of poverty and health outcomes than by temperature or risk of food borne infection. The question therefore remains open, and alternative hypotheses explaining spices’ use pattern, such as conferring protection against cardiovascular disease, neurodegenerative conditions, chronic inflammation obesity, and type 2 diabetes should be explored [10]. In addition, another potential and under investigated explanation is that spice consumption would be an adaptation that prevents cancer. Investigate this hypothesis is important as it may allow evolutionary principles to be applied to slow down or prevent cancer progression [11], for example by increasing spice consumption for at risk patients.

Although a positive association between spicy food intake and cancer risk has been reported in some studies (e.g. [12] and references therein), others conversely suggest that molecules contained in certain spices have the potential to mediate cancer progression, for example by mitigating the effect of oxidative stress and reducing chronic inflammation, both promoter of cancer [1, 6, 13–16]. Even if cancer prevalence is nowadays exacerbated by many components of our modern lifestyle (e.g. smoking, alcohol, environmental pollutants, diet, physical inactivity etc, [17, 18], there is increasing evidence from palaeopathological studies that oncological diseases has accompanied our ancestors during all periods of their evolution, dating back as far as 1.7 Mya ([19], see also [20, 21]). We hypothesize here that spices, depending on their local availability to human populations, may have been incorporated into recipes as a kind of prophylaxis against oncological pathologies. Since most cancers do not show visible symptoms until a certain (often late) threshold in the course of tumorigenesis, spice consumption would not really have evolved as individual decisions made on the basis of health status (i.e. when one is ill), but rather as cultural habits following long-term observations of correlational relationships between specific diets and the risk of premature death (in this case cancer induced) [7, 22]. Even if most human metastatic cancers occur during the post-reproductive period, several reasons suggest that spices may have benefit against cancer [23–25]. First, death induced by cancers is only the final stage of slow and progressive tumorigenic processes whose sub-clinical stages are also likely to be detrimental to fitness [26]. Second, inclusive fitness effects in humans through grandparental care can select for cancer suppression strategy into old age [27]. Finally, while consuming spices could have evolved as an adaptive prophylactic response to supress tumour growth, it could also be the result of cultural evolution with traditions being passed down generations.

To provide additional insights into the debate on the origin of spice use by humans, we used the newly published spice consumption data by Bromham *et al.* [2] and tested, at an international scale, for a potential association between the age standardized incidence rates of gastrointestinal and upper aerodigestive track cancers.

## MATERIAL AND METHODS

### Data collection

#### Age-standardized incidence rates of upper aerodigestive track and gastrointestinal cancers

Age-standardized incidence rates (ASR, all ages and sexes considered and expressed in cases per 100 000 persons) for lips and oral cavity, salivary gland, oesophagus, larynx, oropharynx, stomach, liver, gallbladder, pancreas, colon, rectum and anus cancer were obtained from the International Agency for Research on Cancer database (IARC, https://gco.iarc.fr/) for the year 2020. We focused on organs directly in contact with the spices when food is ingested or directly involved in their digestion. Since there is a lag between the exposure to a cancer risk factor and cancer diagnostic, we obtained data to investigate the effect of a range of risk factor on cancer ASRs for three different years: 2010, 2015 and 2018.

#### Spice consumption per country

**Figure 1.**
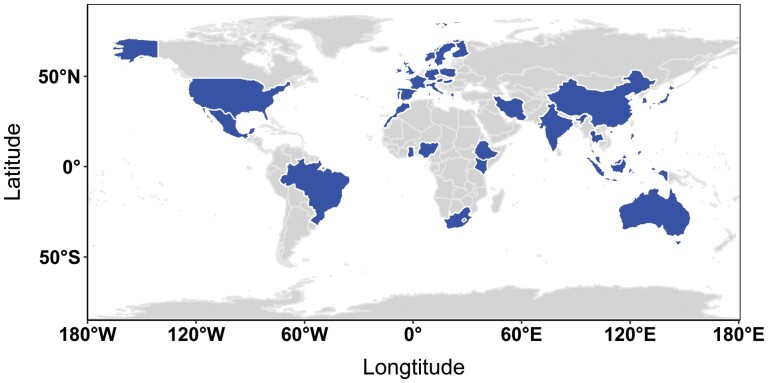
World map with, in blue, the 36 countries included in this study with both spice consumption and cancer data available. Spice data were expressed as the average number of spice ingredients included in a recipe, while the cancer data were expressed in age-standardized incidence rates.

Data on spice consumption was obtained from the study by Bromham *et al.* [2]. The authors of this study constructed a dataset consisting of cuisines from 31 national, 2 super-national and 37 sub-national regions which they aggregated at the national level extending the work of Billing and Sherman [1, 5]. These data were derived from 93 traditional cookbooks from which the number and composition of spice ingredients were extracted (see [2] for full details about their methodology). Billing and Sherman studies did not distinguish between seasonings, condiments or herbs and included spices used in regional cuisines that are not listed in the original Billing and Sherman dataset as long as they found published evidence of their antimicrobial properties (see Supplementary Table S1 for the full list of spices included in this dataset). The study provides the mean number of spices used in all recipes for a given country, with a breakdown of the mean number of species used in meat and vegetable dishes. To build on this original dataset we obtained data from 36 countries for which age-standardized incidence rates of cancer data were available: six African, 11 Asian, 15 European, one North American and one Oceanian (Fig. 1). Following ref. [2], we computed for each country the coordinates (expressed in degrees) of the centroid of the polygons defined by their respective boundaries.

#### Alcohol and tobacco consumption

Alcohol is a well-described risk factor of the upper-aerodigestive track, colon, rectum and liver cancer [28–30] and should therefore be accounted as a confounding factor. We obtained alcohol consumption data, expressed in litres of pure alcohol consumed per capita per year for 2010, 2015 and 2018 from the Our World in Data website (https://ourworldindata.org/).

Tobacco has a synergetic effect with alcohol, significantly increasing the risk of cancer upper aerodigestive and digestive tracts [31, 32]. Data expressed as the age-standardized prevalence of the consumption of tobacco products was obtained for the years, 2010, 2014, 2016 and 2018 from the World Health Organization Global Health Observatory data repository (https://apps.who.int/gho/data/node.main.TOBAGESTDCURR?lang=en). Tobacco data for 2015 was obtained by interpolating the values between the years 2014 and 2016.

#### Human development index

The Human Development Index (HDI) is a measure of human development calculated by combining data on life expectancy, education and living standards. As a country develops (and its HDI increases), the composition of the cancer burden in the population changes. In fact, low-HDI countries are characterized by lower age-standardized incidence rates of stomach, cervical and lung cancer than high-HDI countries. Similarly, high-HDI countries are characterized by a higher incidence of liver, colon, breast and prostate cancer than low-HDI countries [33, 34]. Data were obtained for the year 2010, 2015 and 2018 from the United Nation Development Programme website (http://hdr.undp.org/en/indicators/137506).

### Statistical analyses

We analysed the data using a two-stage protocol. First, we fitted univariate linear models (i.e. without including confounding variables) between the age-standardized incidence rate of each cancer type and the average number of spices included in all recipes, whether meat or vegetable based. Cancer types with a statistically significant slope were then considered to be potentially affected by the average number of spices included in the recipes and were therefore retained for further analysis. Similarly, if the slope of a model was not statistically significant, we considered it unlikely that the average number of spice ingredients included in a recipe would have an effect on cancer incidence. For each univariate model, we calculated the associated *R*^2^ (the proportion of the variance in age-standardized incidence rates captured by the model).

For each cancer type selected in the first stage, we fitted multivariate models at three-time steps using alcohol, tobacco and HDI data from 2010, 2015 and 2018. This allows to account for the confounding effect of these variables. For each of these time steps, we fitted three models, one to study the effect of spices included in all recipes, one for vegetable recipes and one for meat recipes. We then calculated the overall adjusted *R*^2^ for each model but also the partial *R*^2^ for each risk factor. The partial *R*^2^ quantifies the percentage of the remaining variance in age-standardized incidence rates that can be explained by a given risk factor when all other confounding variables have been taken into account. Furthermore we ensured there was no overly high collinearity between the explanatory variables included in all the linear models (max *R*^2^ < 0.40)

Bromham *et al*. showed it is important to investigate for spatial autocorrelation when working with spatial data. Cancer data are also often spatially autocorrelated [11, 35]. Therefore after fitting any multivariate model, we quantified the spatial autocorrelation in the residuals using the Moran’s I (indicative of a potential lack of independence between the age-standardized incidence rates of cancer of various countries). The significance of computed Moran’s I was estimated by a *z*-test using Monte-Carlo draws.

When needed we computed marginal effects to visualize the effect of the average number of spices included in recipes on the age-standardized incidence rate of cancer when confounding variables were accounted for. Marginal effects were determined by computing the prediction of a model by varying one risk factor at time while holding all the other ones constant (here at their average value).

All statistical analyses were performed using R version 4.0.2.

## RESULTS

**Figure 2.**
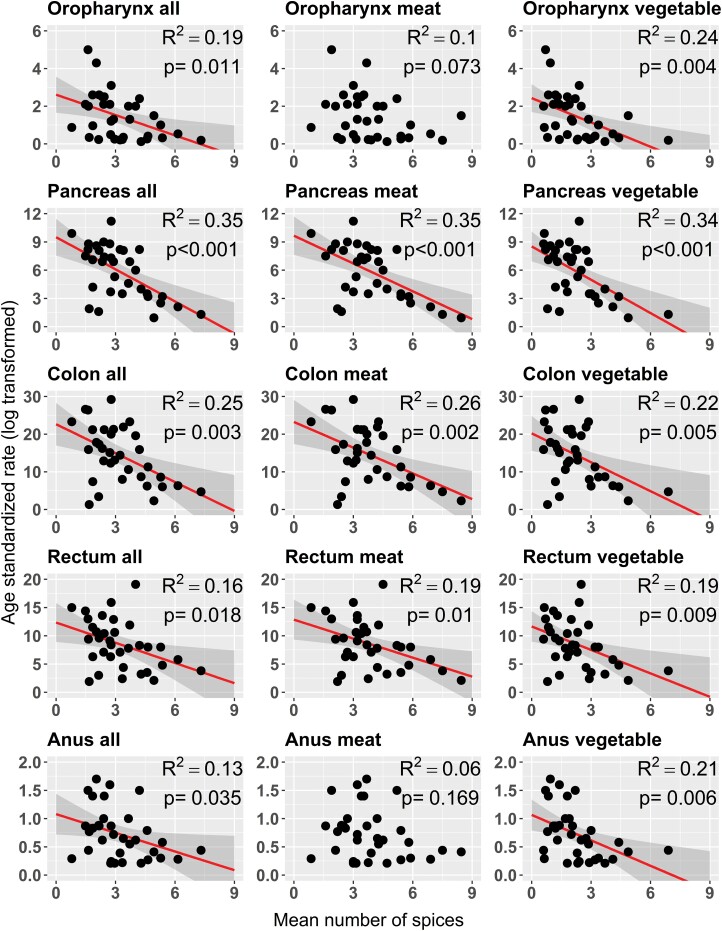
Effect of the mean number of spices included in recipes on the age-standardized rate of gastrointestinal cancers using univariate models (i.e. not accounting for confounding variables). Only cancer types with at least one significant model are presented (all other cancer types are presented in Supplementary data 1). Statistically significant linear regression lines (in red) are showed with their 95% confidence intervals bands (in grey). After controlling for confounding variables in multivariate models, only the relationship between mean number of spices and age-standardized pancreatic cancer rate remained significant.

**Figure 3.**
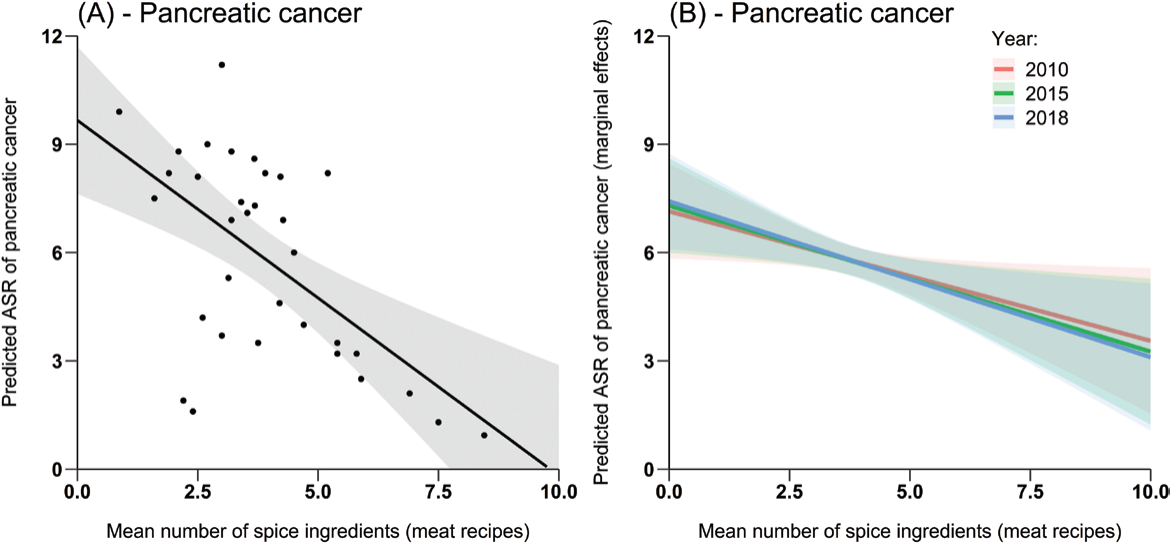
(A) Effect of the mean number of spice ingredients included in meat recipes on the age-standardized incidence rate of pancreas in 36 countries (black dots). (B) Marginal effects of the mean number of spice ingredients included in meat recipes on the age-standardized incidence rates of pancreas cancers when accounting for confounding factors using the 2010, 2015 and 2018 alcohol, tobacco or human development index data. In (A) and (B), the solid lines represent the average predicted by the models and the error bands their associated 95% confidence intervals. Little effect of the year is observed.

The mean number of spice ingredients per recipe was 3.3 ± 1.4 SD for all recipes combined, 3.9 ± 1.7 SD for meat recipes and 2.3 ± 1.4 SD for vegetable recipes. Overall, we found no evidence that spice consumption, expressed as the average number of spice ingredients per recipe, increased or decreased the age-standardized incidence rate of almost all gastrointestinal cancers, regardless of year or recipe type. Five cancer types (oropharynx, pancreas, colon, rectum and anus, Fig. 2) showed a significant relationship with the average number of spice ingredients, but for most of them the observed trend became non-significant when confounding variables (HDI, smoking and alcohol consumption) were taken into account (Supplementary result 1). We only observed a significant reduction in pancreatic cancer rates (Fig. 3). When the average number of ingredients included in meat recipes increased by one, pancreatic cancer rates decreased on average by –0.31 ([95%CI: –0.69, –0.04], *P* = 0.029, overall model-adjusted *R*^2^ = 0.83, partial *R*^2^ = 0.15) when using 2010 data, by –0. 41 ([95%CI: –0.74, –0.09], *P* = 0.0146, multivariate model-adjusted *R*^2^ = 0.82, partial *R*^2^ = 0.18) when 2015 data were used and –0.43 ([95%CI: –0.76, –0.11], *P* = 0.0101, overall adjusted *R*^2^ = 0.81, partial *R*^2^ = 0.20) when 2018 data were used. This potentially suggests a small to moderate protective effect of spices on pancreatic cancer. After controlling for confounders, no significant spatial autocorrelation was observed in the multivariate models (Moran I test, *P* > 0.05).

## DISCUSSION

We found no evidence that spice use affects the age-standardized incidence rate of almost all gastrointestinal cancers in the 36 countries included in our study. Thus, our results do not support the hypothesis that humans have incorporated spices into their diet as a prophylactic against cancers. Our results are also consistent with the findings of Bromham *et al.* [2] that human use of spices is not a mechanism for preventing foodborne disease. If such a mechanism existed, it would logically also affect cancers due to an infectious cause. Indeed, it is estimated that over >20% of the global cancer burden is attributable to infectious agents [36–38], and that a range of food born bacteria are associated with liver, gall bladder and colon cancers [39, 40]. If spices had strong and direct antibacterial activity when added to food, a reduction in cancer rates associated with foodborne pathogens should have been observed as well. However, it is possible that indirect and more difficult to quantify effects on oncogenic processes exist. For example, the consumption of spices is known to have an impact on the gut microbiota, an important immunomodulator against cancer, and may prevent an imbalance in symbiostasis induced by bacterial contamination (through spoiled food). Imbalances in symbiostasis are associated with down-regulation of the anti-cancer immune response [37], and allow for a faster progression of pancreatic cancers [41, 42]. However, the datasets used in this study does not allow to detect such indirect and more subtle effects.

Although we found no evidence that spice consumption affects the risk of most digestive tract cancers internationally, we cannot exclude that this lack of relationship is specifically due to the protective effect of spice consumption in regions with higher cancer risks (e.g. higher risk of foodborne diseases). In this scenario, the protective effect of spices would be responsible for the lack of difference in cancer rates between countries with high and low spice use. This would not be surprising, and a similar phenomenon has already been observed when considering cancer in a wide range of animal species. Indeed, the lack of correlation between animal size and cancer risk is currently attributed to the existence of a higher cancer defence mechanism in larger species (e.g. Peto’s paradox, see [43] and references therein). Our approach of comparing the age-standardized cancer rates between countries is unlikely to demonstrate such effects. A potential way to evaluate the hypothesis that spices’ consumption may have evolved as a local mechanism in front of cancer risk would be to compare cancer rate when related ethnic groups consume, or not, their traditional (spicy) foods (for example using data obtained from diaspora of international communities). Although many confounding factors intervene, several studies have demonstrated that certain ethnic groups, when living outside their historical area, and hence potentially have different dietary habits, have enhanced rate of cancers, or more aggressive ones [44]. In a related vein, it has been suggested that Afro-American in North America have higher cancer rates because their ancestors lived in areas strongly exposed to pathogens, and their locally adapted anti-parasitic adaptations also promote increased proinflammatory immune responses, which in return exacerbate cancers later in life [45]. More generally, there are several evidence of local adaptations in humans that can provide people with protection against factors that are detrimental for the majority of other humans. For instance, Andean populations of the Atacama Desert carries variants of the Arsenic (+3) Methyltrans-Ferase Enzyme (AS3MT) able to metabolize high concentration of arsenic in drinking water [46]. Therefore, we cannot totally exclude at the moment that spices’ consumption could provide some protection against cancer in populations experiencing, for different reasons, higher cancer risks. Further studies would be needed to clarify this point and will require data on spice consumption at a sub-national scale and matching the areas inhabited by those ethnies.

Our results suggest a possible protective effect of spice added to meat recipes on pancreas cancer. However, it remains difficult at the moment to determine whether this link is causal or due to a spurious correlation. For instance, red meat consumption especially when cooked at high temperatures increases the risk of pancreas cancers [47]. If the spices’ consumption is increased in countries having the lowest rate of red meat consumption, a negative relationship may appear but without indicating a causal link. Other potential confounding factor including obesity, family history (i.e. genetic factor) but also occupational exposures (involving exposure to metalworking and pesticides), and allergies were not accounted for in this study [48]. Further studies combining health and spice consumption data at the individual level or over smaller geographical areas are required to explore the role of potentially confounding factors. It is also worth mentioning that the number of countries with a relatively high average number of spice ingredient per recipe is relatively small which may bias the regression models for those values. It is possible that the effects observed in our study weakens as more countries with relatively high spice consumption are included. In addition, as the sample size and spatial coverage increase, we recommend to quantify and control for collinearity between risk factor and spatial-autocorrelation which may bias the model estimates (as recently highlighted by Bromham *et al.* [2], with the patterns of spice consumption and risk of food borne diseases).

In our analyses, we used the average number of spices included in the traditional recipes of each country as a proxy for the typical spice consumption of the inhabitants of these countries. We cannot rule out that at the individual level, consumption of large amounts of a given spice promotes or prevents cancer formation. For example, there is evidence that consumption of large amounts of chili pepper is associated with a higher risk of developing stomach cancer [12, 49, 50] while the consumption of radish and green chili may reduce the risk of gallbladder cancer [50]. In addition, a recent study suggests that increased frequency of consumption of spicy meals is associated with a lower risk of oesophageal and rectal cancers in China. However, the study does not provide a breakdown of the type and amount of spices consumed [14]. Similarly, our study does not consider the potential synergistic effect of multiple spices. Individual data, rather than national average data, including consumption frequencies, doses, and whether spices, are used fresh or powdered also appear to be important factors and would be needed to properly test these effects and their synergies.

In conclusion, our results do not support the hypothesis that spice consumption is an adaptive response to prevent cancer development. Because of the limitations mentioned above, this topic would however deserve deeper investigations.

## Supplementary Material

eoac040_suppl_Supplementary_Material

eoac040_suppl_Supplementary_Data
