# Supplementary material for: No evidence that spice consumption is a cancer prevention mechanism in human populations
Source: Evol Med Public Health. 2022 Nov 24;11(1):45–52. doi: 10.1093/emph/eoac040 (PMC10024787; doi:10.1093/emph/eoac040)
Supplement: eoac040_suppl_Supplementary_Material [file eoac040_suppl_supplementary_material.docx]

**Supplementary material:**



**Supplementary Figure 1:** Effect of the mean number of spices on the age-standardised incidence rate of gastrointestinal cancers investigated using univariate models (i.e. not accounting for confounding variables).

**
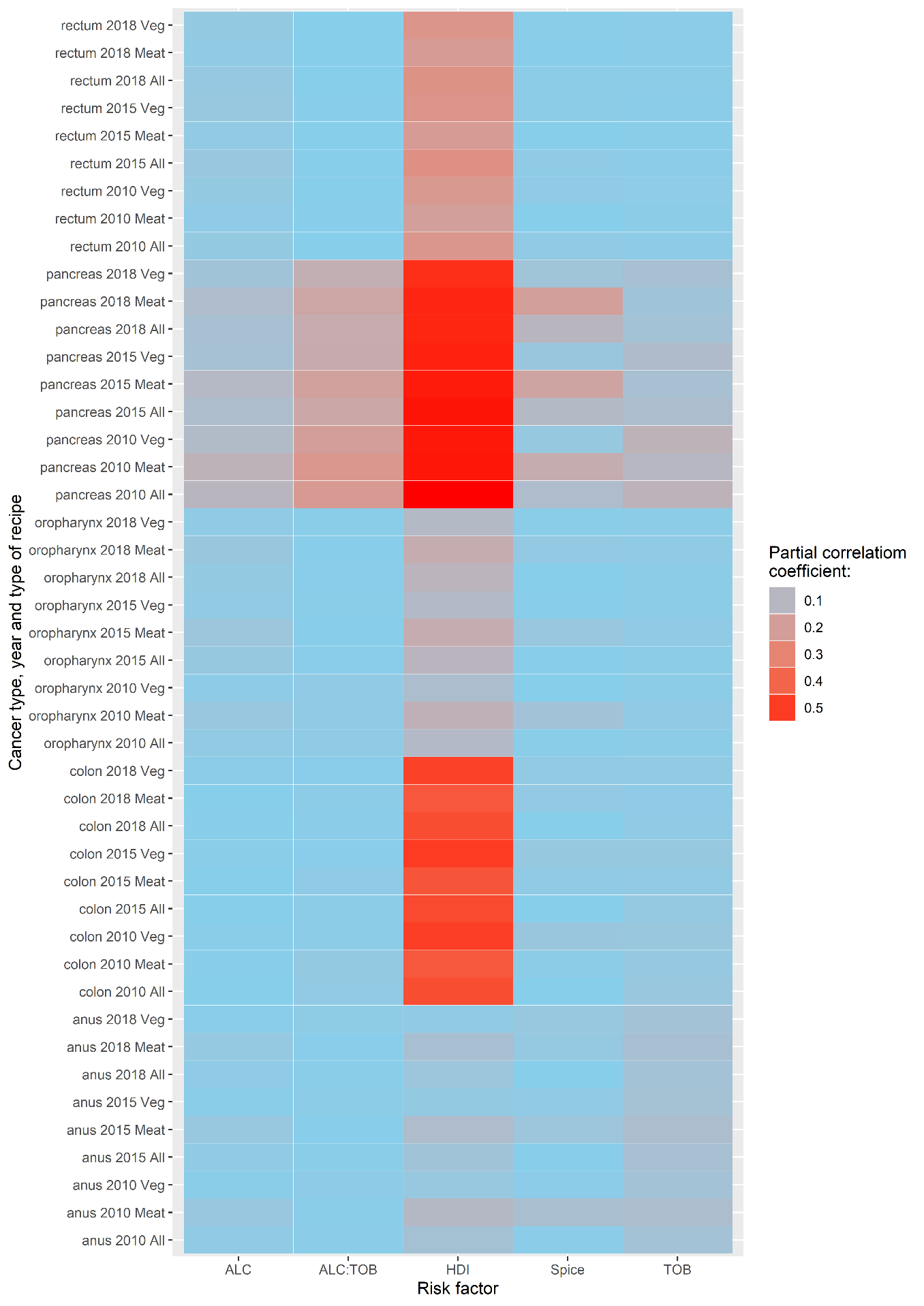
Supplementary Figure 2:** Partial correlation effects for multivariate linear models investigating the effect of the average number of spices ingredients included in a recipe, while accounting for confounding variables (alcohol [ALC], tobacco [TOB], human development index [HDI]). Three years (2010,2015 and 2018) and three different types of recipes (all, meat and vegetable) are considered. Only the pancreatic cancer models using meat recipe data remains significant after accounting for confounding variables.

**Supplementary Table 1:** List of spices ingredients included in the calculation of the average number of spices used in recipes by Bromham et al. 2021.

**Ajwain:** Carom, fruit of *Trachyspermum ammi*

**Allspice**: Dried berries of *Pimenta dioica*

**Amaranth:** Leaves or seeds of *Amaranthus*

**Amchoor:** Dried unripe mango, *Mangifera*

**Anise:** Aniseed, *Pimpinella anisum*

**Anjelica:** Leaves or roots of *Angelica archangelica*

**Artemisia:** Wormwood, *Artemisia annua*

**Asafoetida:** Hing powder, dried gum of *Ferula*

**Basil:** Leaves of *Ocimum*

**Bay:** Leaves of *Laurus nobilis*

**Camphor:** Substance from *Cinnamomum camphora*

**Capers:** Pickled fruit or flowers of *Capparis spinosa*

**Capsicum:** Bell peppers, Capsicum annum or *Capsicum grossum*

**Caraway:** Fruits of *Carum carvi*

**Cardamom:** Seeds of *Elleteria or Amomum*

**Celery**: Seeds, stalks or leaves of *Apium graveolens*

**Chilli:** Small spicy *Capsicum*, including cayenne

**Chamomile:** Flower of *Chamaemelum nobile*

**Chervil:** Leaves of *Anthriscus cerefolium*

**Chrysantheum:** Flower of *Chrysantheum sp.*

**Cinnamon:** Bark of *Cinnamomum*

**Citrus:** All citrus including juice, peel, oil or extract

**Cloves:** Flowers of *Syzygium aromaticum*

**Coriander:** Leaves, seeds or roots of *Coriandrum sativum*

**Cumin:** Seeds of *Cuminum cyminum*

**Curry:** leaf Leaves of *Murraya koenigii*

**Dill:** Leaves or seeds of *Anethum graveolens*

**Drumstick leaves:** Leaves of *Moringa oleifera*

**Epazote:** Leaves of *Levisticum officinale*

**Fennel:** Seeds of *Foeniculum vulgare*

**Fenugreek:** Seeds and leaves of *Trigonella foenum graecum*

**Fuki:** Giant butterbur, *Petasites japonicus*

**Galangal:** Laos, root of *Alpina*

**Garlic:** Roots and shoots of *Allium sativum*

**Garcinia indica:** Kokum, dried fruit of *Garcinia indica*

**Gardeni**a: Seed of Gardenia augusta or *Gardenia jasminoides*

**Ginger:** Root of *Zingiber officinale*

**Hemp:** Seed or oil of *Cannabis sativa*

**Horseradish:** Root of *Armoracia rusticana*

**Hyssop:** Leaves or oil extract from *Hyssopus officinalis*

**Japanese horseradish:** Wasabi, *Eutrema (Wasabia) japonica*

**Japanese parsley:** Leaves of *Oenanthe javanica*

**Japanese pepper:** Sansho, *Zanthoxylum piperitum*

**Juniper:** Berries of *Juniperu*s

**Lavender:** Flowers of *Lavandula*

**Lemon balm:** Leaves of *Melissa officinalis*

**Lemon verbena:** Leaves of *Lippia citriodora*

**Lemongrass:** Leaves or oil of *Cymbopogon*

**Licorice:** Root of *Glycyrrhiza glabra* or *Glycyrrhiza uralensis*

**Lilium:** Bulb or root of *Lilium*

**Long pepper:** Fruit of *Piper longum*

**Lovage:** Seeds or leaves of *Levisticum officinale*

**Marjoram:** Leaves of *Origanum majorana*

**Mastic:** Resin of *Pistacia lentiscus*

**Mate**: Leaves of *Ilex paraguariensis*

**Mexican oregano:** Leaves of *Lippia graveolens*

**Mint:** Leaves of *Mentha*

**Myoga:** Japanese ginger, *Zingiber mioga*

**Mitsuba:** Leaves of *Cryptotaenia*

**Mountain pepper:** Fruit of *Lindera glauca*

**Mustard:** Seeds or oil of *Brassica alba, Brassica juncea* or *Brassica nigra*

**Nigella:** Seeds of *Nigella sativa*

**Nutmeg:** Fruit of *Myristica fragrans* including mace

**Onion *Allium*:** includes chives, leeks, shallots

**Oregano:** Leaves of *Origanum vulgare*

**Paprika:** Dried, powdered *Capsicum annum*

**Parsley:** Leaves of *Petroselinum crispum*

**Peony:** Root of Paeonia (paeonol)

**Pepper:** Black and white pepper, *Piper nigrum*

**Peppermint:** Leaves of *Mentha piperita*

**Perilla:** *Perilla frutescens var. crispa*

**Poppy:** Seeds of *Papaver somniferum*

**Pomegranate:** Including anaardana (dried pomegranate seeds)

**Radish:** Roots of Raphanus, including daikon (*Raphanus sativus*), mu (*Raphanus raphanistrum*)

**Rose:** Extract from flowers or buds of *Rosa indica*

**Rosemary:** Leaves of *Rosmarinus officinalis*

**Sage:** Leaves of *Salvia officinalis*

**Saffron:** Flower of *Crocus sativus*

**Sandalwood:** Extract from wood of *Santalum sp.*

**Savory:** Leaves of *Satureja*

**Seaweed:** Includes kelp, nori, carageenen

**Sesame:** Seeds and oil from *Sesamum indicum*

**Sichuan pepper:** *Zanthoxylum simulans* or *Zanthoxylum bungeanum*

**Star anise:** *Illicium verum*

**Tamarind:** Fruit of *Tamarindus indica*

**Tarragon:** Leaves of *Artemisia dracunculus*

**Te**a: Leaves or oil of *Camellia sinensis*

**Thyme:** *Leaves of Thymus*

**Toon:** Chunya, *Toona sinensis*

**Tumeric:** Root of *Curcuma longa*

**Vanilla:** Fruit, oil or extract of Vanilla

**Vetiver:** Extract or oil from *Chrysopogon zizanioides*

**Wild tumeric:** Root of *Curcuma aromatica*
